# Supplementary material for: A robust normalized local filter to estimate compositional heterogeneity directly from cryo-EM maps
Source: Nat Commun. 2023 Sep 19;14:5802. doi: 10.1038/s41467-023-41478-1 (PMC10509264; doi:10.1038/s41467-023-41478-1)
Supplement: Supplementary file 1 — Supplementary Information [file 41467_2023_41478_MOESM1_ESM.pdf]

Supplementary information to "A robust normalized local filter to estimate compositional heterogeneity directly from cryo-EM maps"

Bjoern O. Forsberg [1,2,✉], **Pranav N.M. Shah** [2], **Alister Burt**[3]

[1]Department of Physiology and Pharmacology, Karolinska Institute, 171 77 Stockholm, Sweden. ; [2]Division of Structural Biology, University of Oxford, OX3 7BN Oxford, UK; [3]MRC Laboratory of Molecular Biology, Cambridge CB2 0QH, UK.

Map	Size	ResMap	RELION	LocOcc.	OccuPy
EMD-33442	200	172	6’300	480	4
EMD-12104	300	572	79’660	828	6
EMD-7770	338	335	25’770	1’092	10
EMD-13015	420	886	449’260	696	11

Supplementary Table 1. The execution time of local property estimation by ResMap [44], RELION [13], and LocOccupancy [50]. Reported time is in CPU-seconds, as RELION and LocOccupancy execute with process-level parallelism.

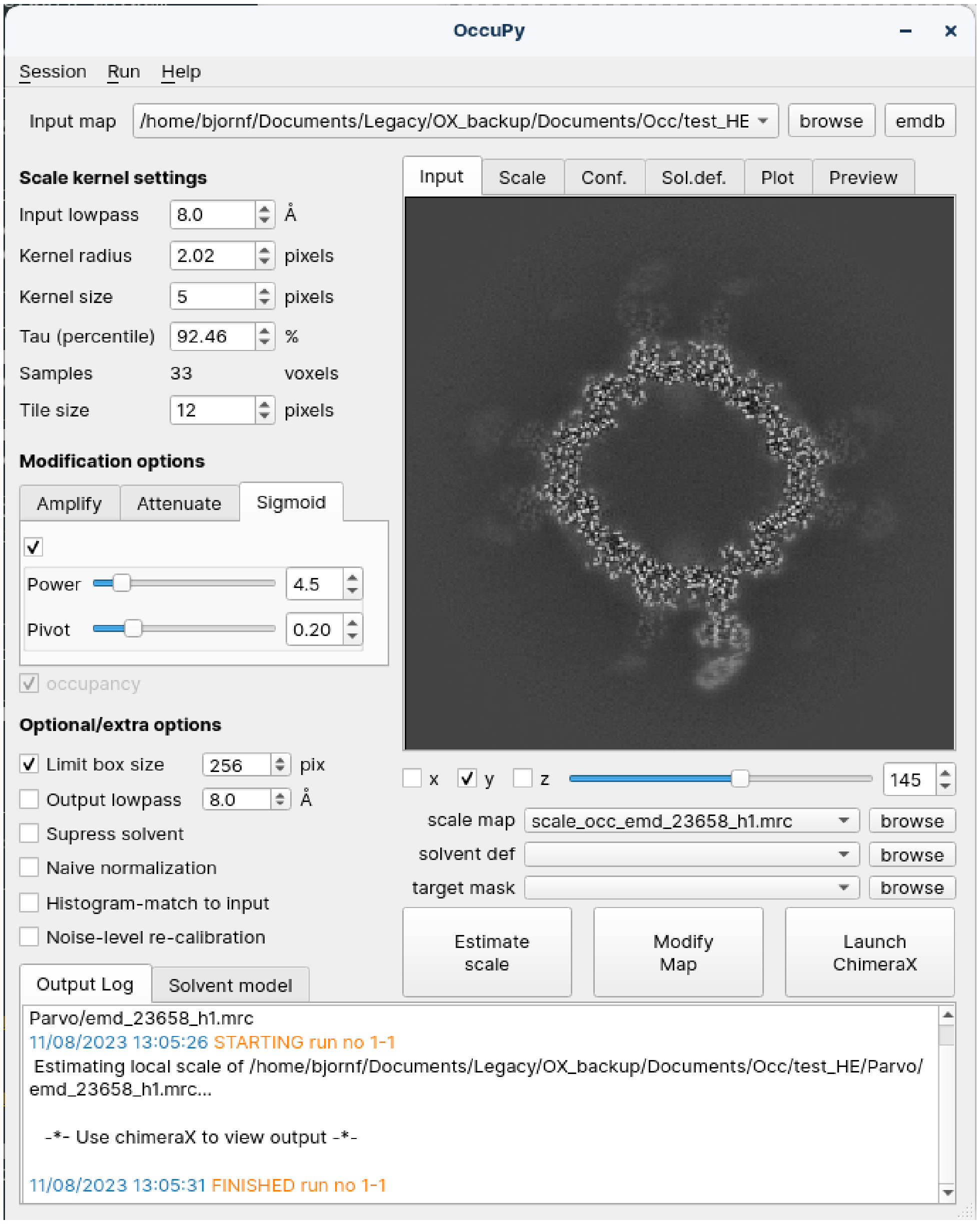

Supplementary Figure 1. The OccuPy GUI. The GUI is written in PyQt5, using the python libraries mrcfile [55], numpy [56], scipy [57], and matplotlib [58]. The user may open and visualize the input map, modify the automatically generated kernel settings, and estimate local scale. The scale, solvent model, and confidence can be directly inspected, and settings adjusted. Modifications can also be previewed in real-time, and generated with additional output control. An exhaustive log documents processing parameters is also maintained. Annotated visualization of estimated scale and modified maps is facilitated through a command script for UCSF ChimeraX [53].

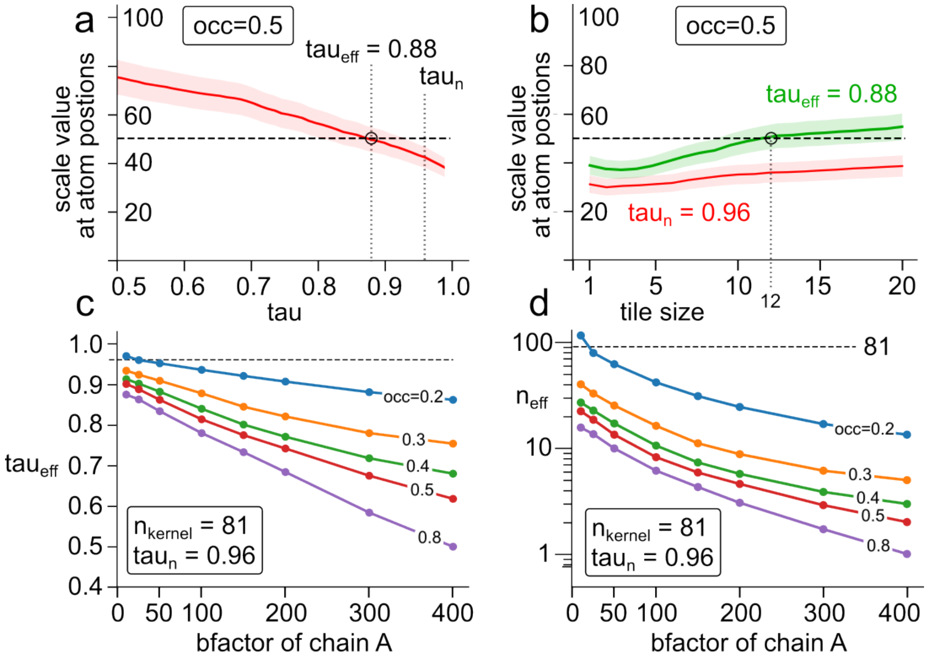

Supplementary Figure 2. Input parameter dependence of local scale estimate. a, Occupancy-mode local scale determined as a function of normalization tile percentile τ, against synthetic data with one full-occupancy chain and evaluated at a chain of designated occupancy occ=0.5, and default tile-size of 12^3 voxels. **b**, Occupancy-mode local scale determined as a function of normalization tile size, at the theoretically derived value of $\tau = \tau_{\mathrm{n}}$ as well as at the value τ_n which optimizes occupancy estimate at default tile size. **c**, Estimated optimal value of $\tau = \tau_{\mathrm{eff}}$ as dependent on chain occupancy and bfactor. **d**, Effective sampling number n_{eff} corresponding to the optimal value of $\tau = \tau_n$, as dependent on chain occupancy and bfactor.

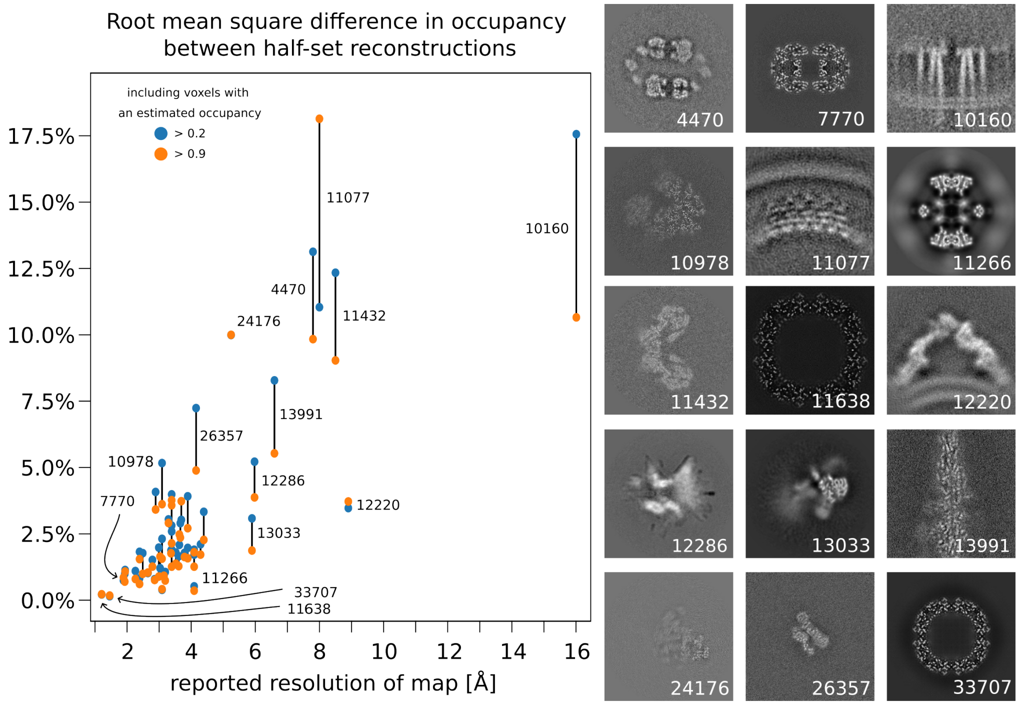

Supplementary Figure 3. Difference in local scale estimated from half-set reconstructions. The RMS of the occupancy was calculated using all voxels with an occupancy above 0.2 (blue) and 0.9 (orange), plotted as dependent on the reported resolution. Volume slice representation is also shown for all points labeled by their EMDB entry.

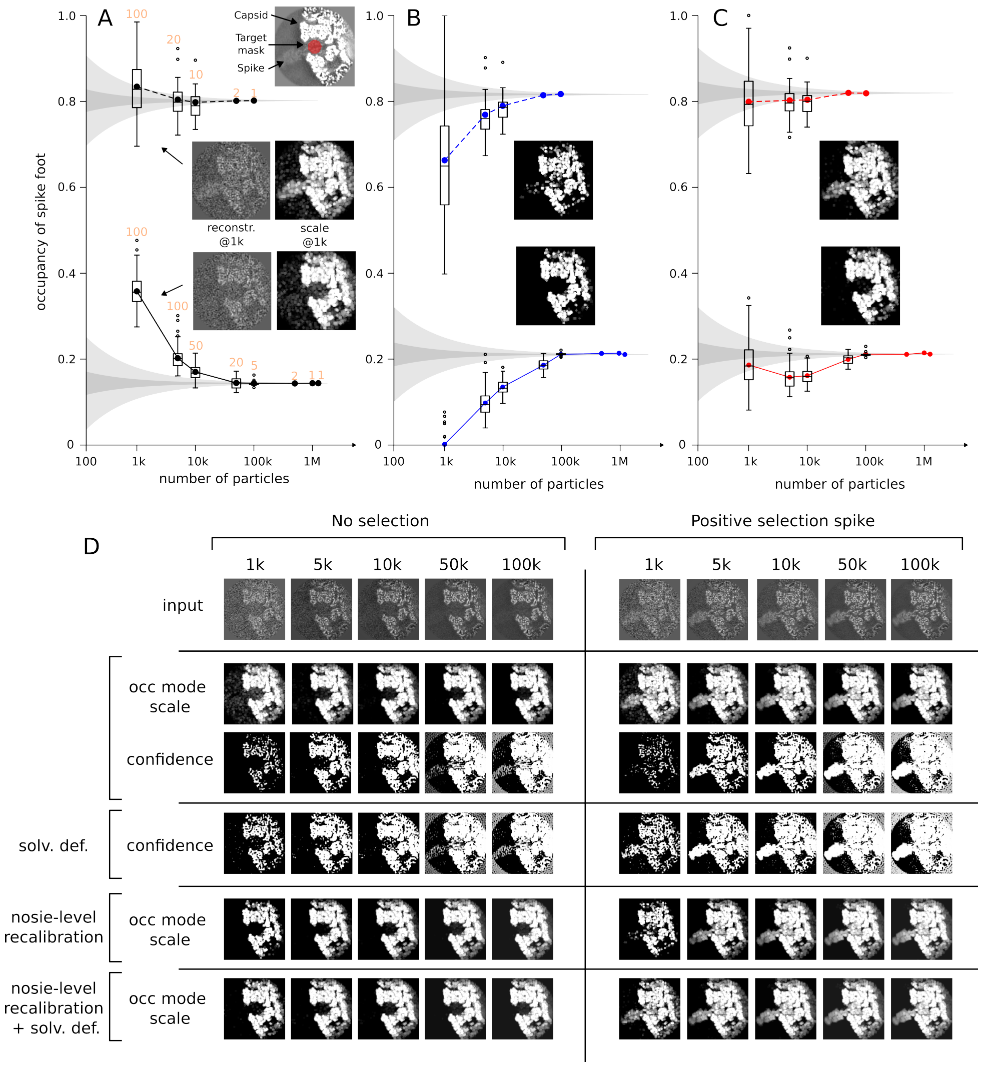

Supplementary Figure 4. Evaluation of occupancy in the presence of real data with noise. a Estimated occupancy of rotavirus spike protein, as a function of number of particles used for the 3D reconstruction, and thus the level of noise. The solid line represents that of a random subsets of the input data without replacement, and the dashed line the same drawn from a set of images separated by positive selection of the spike by conventional classification in RELION. Dark grey intervals indicate the expected inter-quartile range of an averaged Bernoulli random variable at the asymptotic value, and in light grey the correspondingly expected whiskers. An inset also shows an example volumetric slice of the reconstructions used at 1k particles, along with the corresponding estimated occupancy mode scale. The number of separate subsets examined at each sample size are indicated in orange at each given value. **b** As in panel a, using noise-level recalibration as described in methods.. **c** As in panel b, having additionally provided a solvent definition that delineates a region of solvent noise. **d** Example reconstructions, occupancy mode local scale, and confidence under the examined conditions, across the tested particles numbers up to 100k particles. Note that the addition of a solvent definition without noise-level correction does not alter the estimated scale, but only the confidence. Similarly, noise-level correction does not alter the confidence.

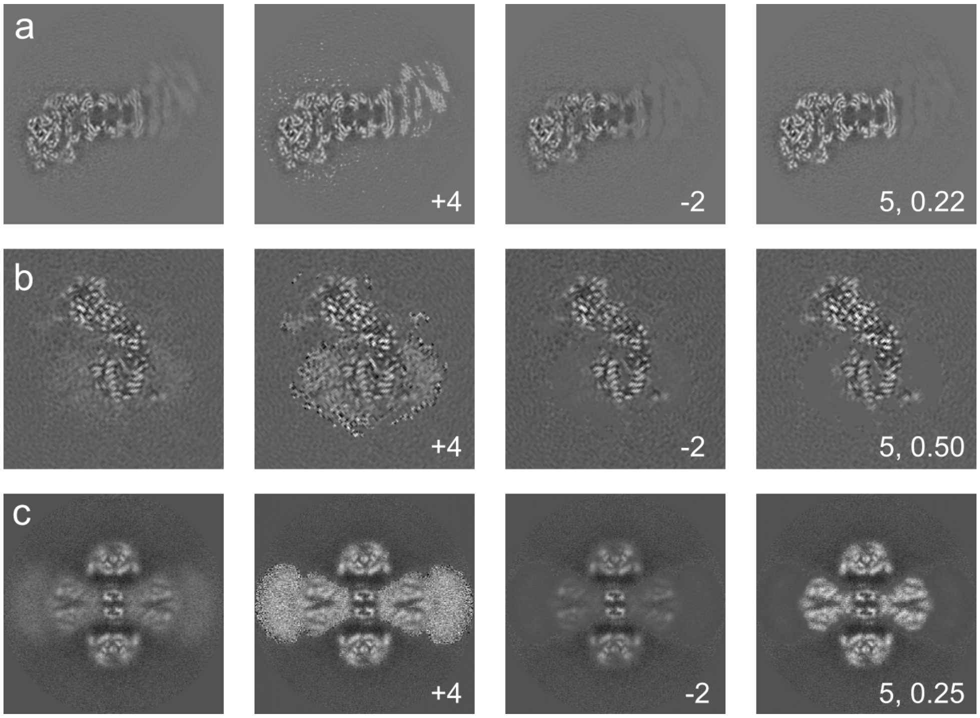

Supplementary Figure 5. Central slices of modified reconstructions. a, EMD-14085 shown as a central slice. Further, it was amplified ($\gamma = 4$), attenuated ($\gamma = 2$), and sigmoid-modified ($\gamma = 5, \mu = 0.22$). **b**, EMD-3061, as in panel a, with sigmoid pivot $\mu = 0.5$. **c**, EMD-31466, as in panel a, with sigmoid pivot $\mu = 0.25$.

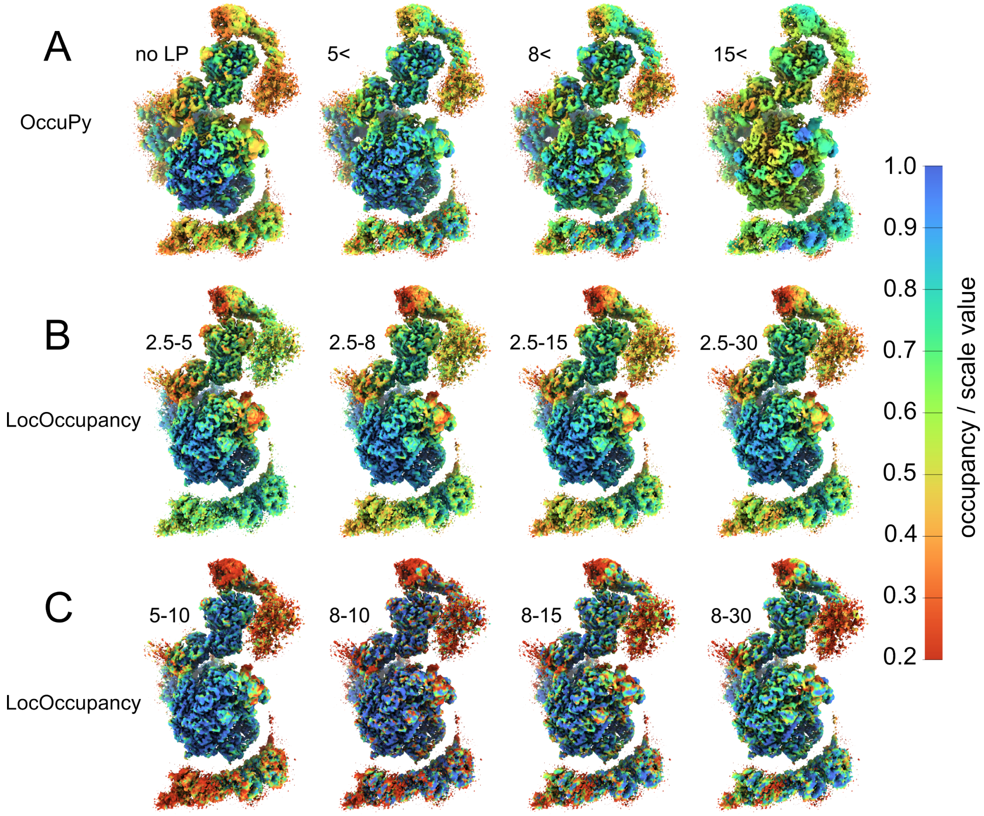

Supplementary Figure 6. Comparison with LocOccupancy. EMD-13015 was used to estimate the influence of differential resolution on local occupancy estimation, as it depends on the resolution-mitigating low-pass (LP) filter in OccuPy and the range of resolutions considered in LocOccupancy, respectively. **a**, occupancy-mode local scale as estimated by occupy, utilizing LP to the designated resolution in Å. **b-c**, Occupancy estimated by locOccupancy using the designated range of resolution in Å.

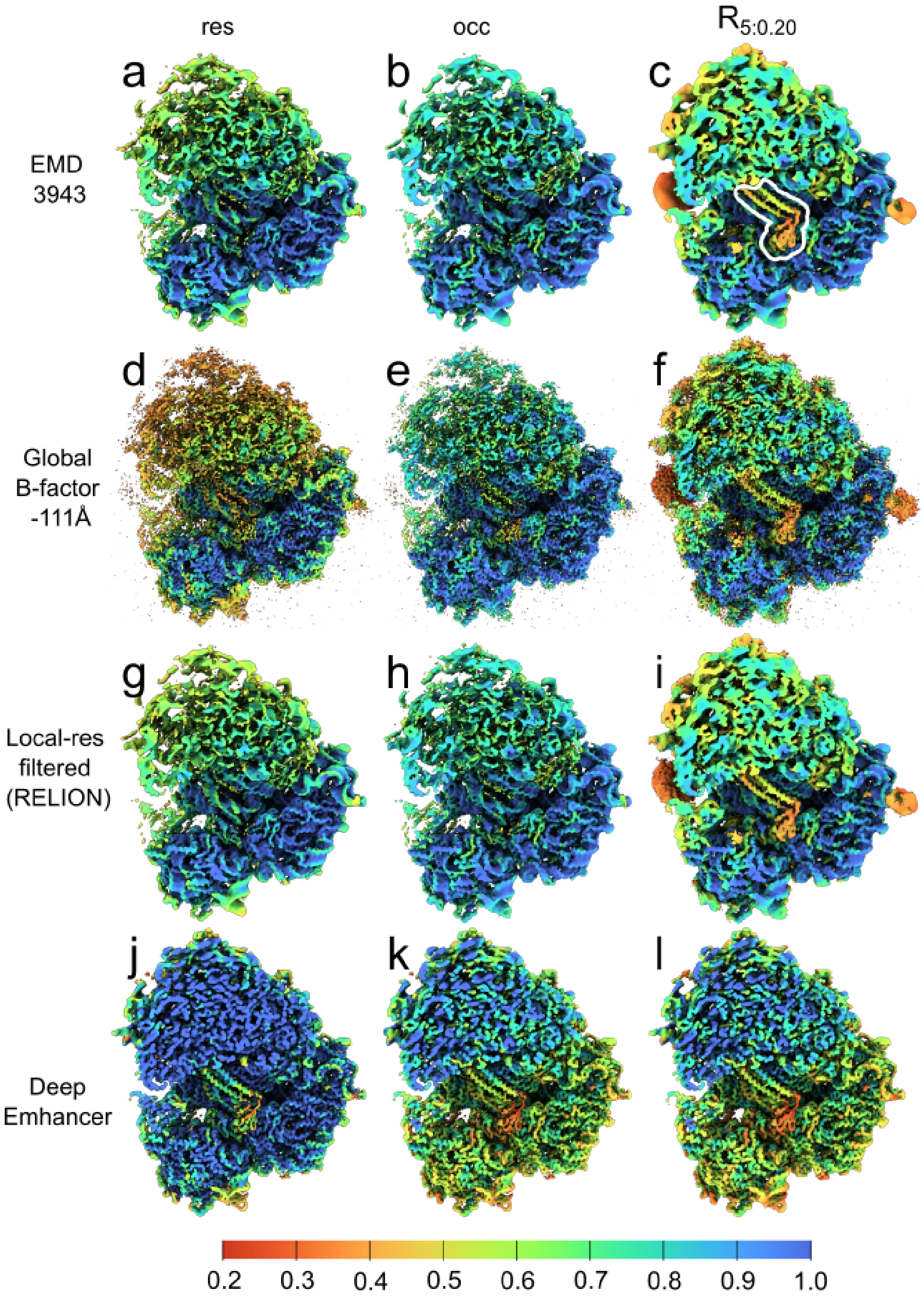

Supplementary Figure 7. Local scale of modified maps. All views are cutaway. **a-b**, EMD-3943 colored according to the estimated local scale and occupancy-mode local scale, respectively. **c**, EMD-3943 sigmoid-modified with power $\nu = 5$, and pivot $\mu = 0.2$, using the estimated occupancy-mode scale. The RRF is circles in white outline for clarity. **d-f**, as panels **a-c** following global B-factor sharpening in RELION [13]. **g-i**, as panels **a-c** following local FSC-filtering in RELION [13]. **j-l**, as panels **a-c** following sharpening by the neural-net based DeepEMhancer [50].

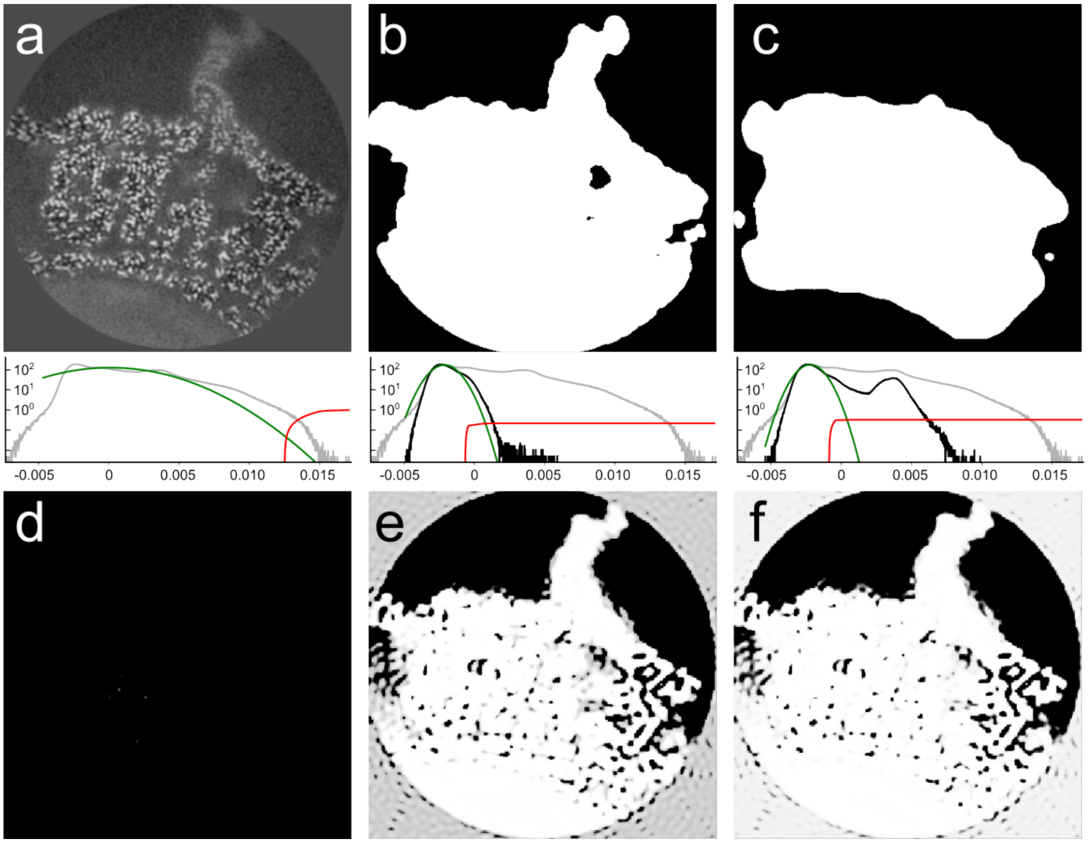

Supplementary Figure 8. The use of a solvent definition to aid solvent model estimation. a, The reconstruction of an asymmetric unit of a viral capsid is re-framed within the original reconstruction, with a very small fractional volume of solvent, leading to an overestimated solvent variance. The solvent model is shown in green, the confidence in red, and the histogram of the data used to establish the solvent model is shown in black. The histogram of the input map is shown in gray. **b**, An accurate solvent definition and the resulting solvent model. **c**, A less accurate solvent definition. **d**, The estimated confidence without a solvent definition. **e**, The estimated confidence with the accurate solvent definition in panel **b**. **f**, The estimated confidence with the less accurate solvent definition in panel **c**.

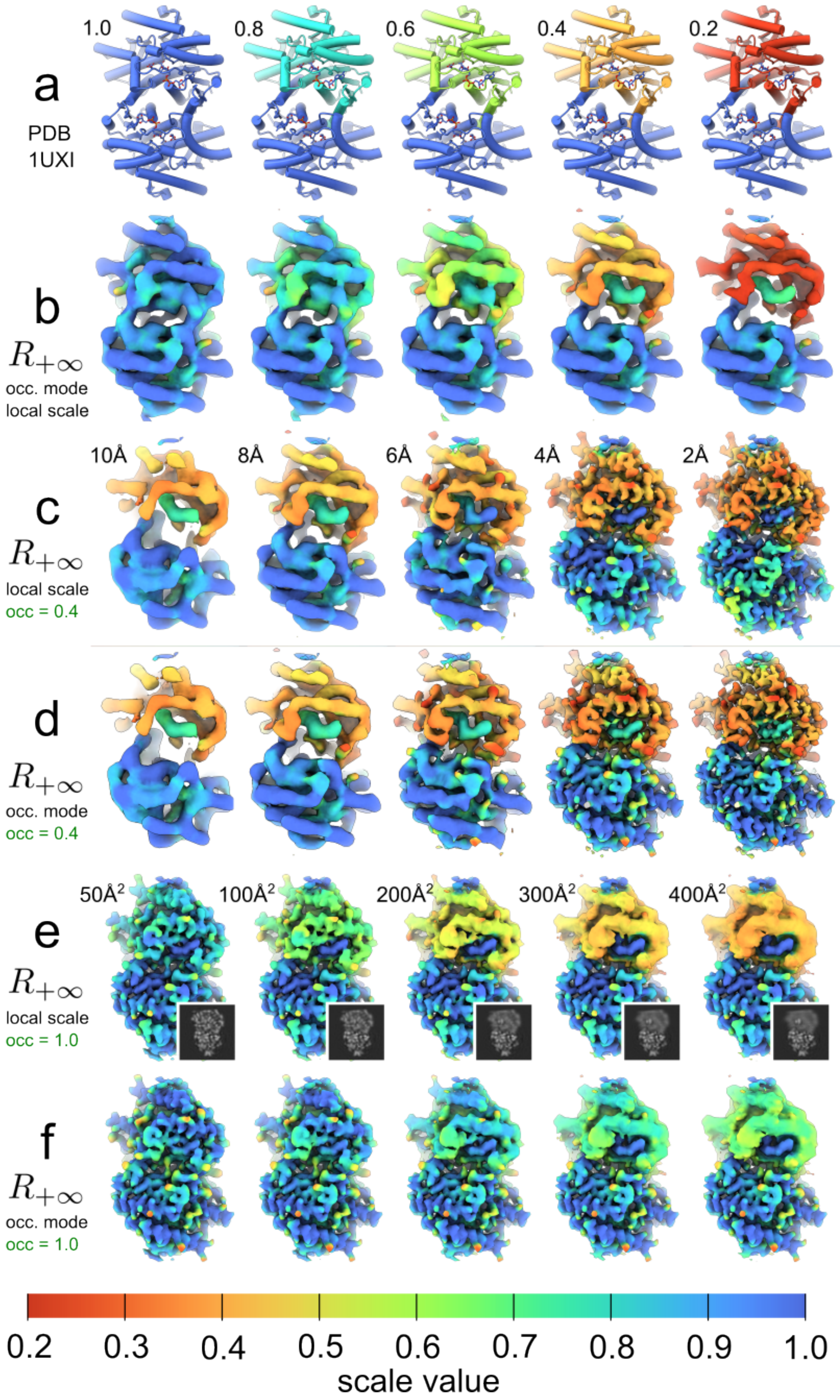

Supplementary Figure 9. Evaluation of local scale against synthetic data. All shown densities are fully amplified and colored according to the indicated local scale. **a**, The occupancy field of all atoms in chain A of the malate dehydrogenase dimer PDB-1uxi structure file was altered to reflect the indicated occupancy. Chain B, and the two nicotinamide adenine-dinucleotide (NAD) cofactors were left at full occupancy (1.0). Theoretical densities at 8Å resolution were generated from the atomic model using the program gemmi. **b**, Occupancy-mode local scale at various theoretical occupancies and 8Å resolution density. **c**, Local scale at fixed partial occupancy of chain A, at various generated map resolutions. **d**, Occupancy-mode as in panel **c**. **e**, Local scale after altering B-factors of chain A as indicated, leaving occupancy of all atoms at 100%. **f**, Occupancy-mode as in panel **e**.

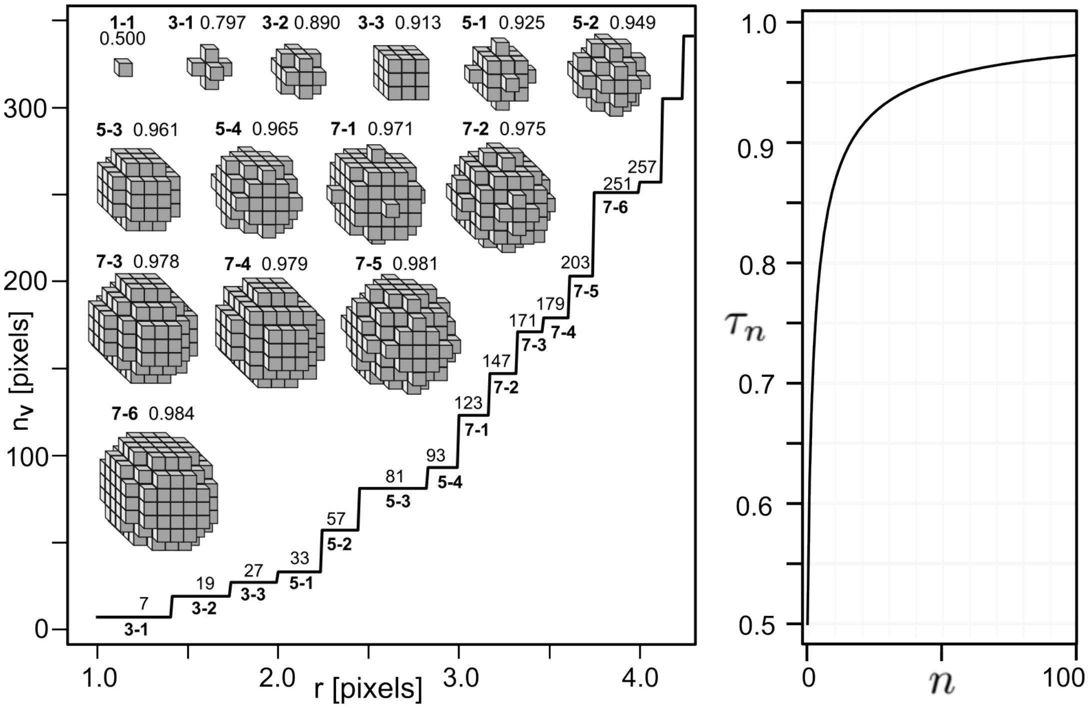

Supplementary Figure 10. Radial cutoff kernels used in OccuPy. The kernels are numbered by kernel size and order. The number of sampling points within the kernel is plotted. Further to this, the value of τ_n is plotted for a number of sampling size n. Manually altering automatically derived parameters may cause less spherical kernels in some cases, e.g. a 5x5x5.

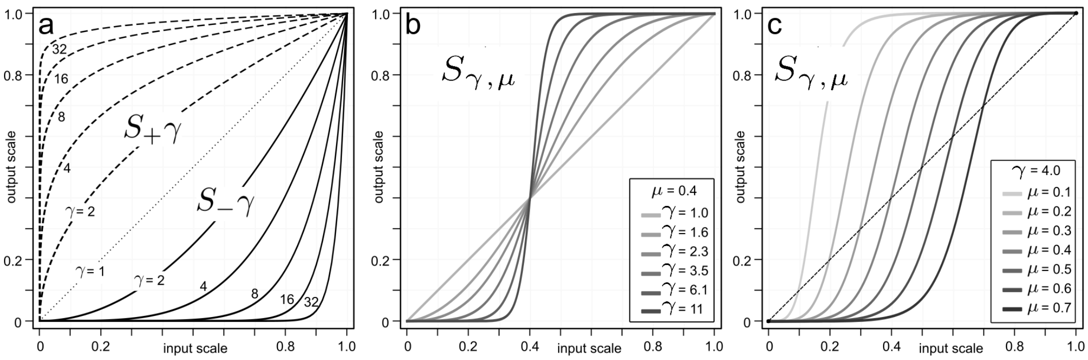

Supplementary Figure 11. Mapping of modified scale. **a**, Amplification and attenuation of estimated map scale by the power coefficient γ. **b**, Sigmoid modification, at variable power γ but fixed pivot μ. **c**, Sigmoid modification, at fixed power γ and variable pivot μ. $S_{\gamma,\mu}$

Supplementary Note 1

EMDB entries used for development

Entries in bold are used in figures within the paper. One of the deposited half maps was used for all entries where this was available, since deposited "main" maps can be post-processed, masked, and/or a composite reconstruction without disclosure, at the discretion of the depositor.

- EMD-0056
- EMD-0355
- EMD-0452
- EMD-0667
- EMD-0910
- EMD-3061
- **EMD-3943**
- EMD-2984
- EMD-4058
- EMD-4470
- EMD-5529
- EMD-6840
- EMD-7770
- EMD-8194
- EMD-8332
- EMD-8333
- EMD-9108
- EMD-09215
- EMD-10160
- EMD-10978
- EMD-11077
- EMD-11266
- EMD-11268
- EMD-11432
- EMD-11576
- **EMD-11638**
- **EMD-12104**
- EMD-12112
- EMD-12197
- EMD-12202
- EMD-12204
- EMD-12220
- EMD-12286
- EMD-12341
- EMD-12870
- EMD-12885
- **EMD-13015**
- EMD-13033
- EMD-13078
- EMD-13162
- EMD-13163
- EMD-13241
- EMD-13378
- **EMD-13611**
- EMD-13619
- EMD-13620
- EMD-13886
- EMD-13961
- EMD-13991
- **EMD-14085**
- EMD-14122
- EMD-14336
- EMD-14369
- EMD-14428
- EMD-20546
- EMD-21485
- EMD-21847
- EMD-22074
- EMD-22075
- EMD-22358
- EMD-22855
- EMD-22959
- EMD-23006
- EMD-23015
- EMD-23017
- EMD-23082
- EMD-23096
- EMD-23099
- EMD-23114
- EMD-23121
- EMD-23658
- EMD-24095
- EMD-24176
- EMD-24536
- EMD-24717
- EMD-24823
- EMD-24976
- EMD-25121
- EMD-25170
- EMD-26357
- EMD-26477
- EMD-26513
- **EMD-30171**
- EMD-30547
- EMD-30675
- EMD-31213
- EMD-31432
- **EMD-31466**
- EMD-32121
- EMD-32126
- **EMD-32148**
- EMD-32593
- EMD-32612
- EMD-32875
- EMD-33707
- EMD-34028
